# Bariatric surgery reduces CD36-bearing microvesicles of endothelial and monocyte origin

**DOI:** 10.1186/s12986-018-0309-4

**Published:** 2018-10-23

**Authors:** Jaco Botha, Morten Hjuler Nielsen, Maja Høegh Christensen, Henrik Vestergaard, Aase Handberg

**Affiliations:** 1 Department of Clinical Biochemistry, Aalborg University Hospital, Hobrovej 18-22, DK-9000 Aalborg, Denmark; 2 Department of Clinical Medicine, Faculty of Medicine, Aalborg University, Sdr. Skovvej 15, DK-9000 Aalborg, Denmark; 3 Novo Nordisk Foundation Center for Basic Metabolic Research, Section of Metabolic Genetics, SUND, University of Copenhagen, Panum, Mærsk tårnet, Bygning 7, 8. Etage, DK-2200 Copenhagen N, Denmark

**Keywords:** Extracellular vesicles, CD36, Obesity, Ectopic fat deposition, Metabolic syndrome, Bariatric surgery

## Abstract

**Background:**

Bariatric surgery is a widely adopted treatment for obesity and its secondary complications. In the past decade, microvesicles (MVs) and CD36 have increasingly been considered as possible biomarkers for obesity, the metabolic syndrome (MetSy), type 2 diabetes mellitus (T2DM). Thus, the purpose of this study was to investigate how weight loss resulting from bariatric surgery affects levels of specific MV phenotypes and their expression of CD36 scavenger receptor. Additionally, we hypothesised that subjects with MetSy had higher baseline concentrations of investigated MV phenotypes.

**Methods:**

Twenty individuals undergoing *Roux-en-Y* gastric bypass surgery were evaluated before and 3 months after surgery. MVs were characterised by flow cytometry at both time points and defined as lactadherin-binding particles within a 100-1000 nm size gate. MVs of monocyte (CD14) and endothelial (CD62E) origin were defined by cell-specific markers, and their expression of CD36 was investigated.

**Results:**

Following bariatric surgery, subjects incurred an average BMI reduction (delta) of − 8.4 ± 1.4 (*p* < 0.0001). Significant reductions were observed for the total MVs (− 66.55%, *p* = 0.0017) and MVs of monocyte (− 36.11%, *p* = 0.0056) and endothelial (− 40.10%, *p* = 0.0007) origins. Although the bulk of CD36-bearing MVs were unaltered, significant reductions were observed for CD36-bearing MVs of monocyte (− 60.04%, *p* = 0.0192) and endothelial (− 54.93%, *p* = 0.04) origin. No differences in levels of MVs were identified between subjects who presented with MetSy at baseline (*n* = 13) and those that did not (*n* = 7).

**Conclusion:**

Bariatric surgery resulted in significantly altered levels of CD36-bearing MVs of monocyte and endothelial origin. This likely reflects improvements in ectopic fat distribution, plasma lipid profile, low-grade inflammation, and oxidative stress following weight loss. Conversely, however, the presence of MetSy at baseline had no impact on MV phenotypes.

**Electronic supplementary material:**

The online version of this article (10.1186/s12986-018-0309-4) contains supplementary material, which is available to authorized users.

## Background

Decades of research indicate that lifestyle interventions and pharmacotherapy of obesity often fail to result in sufficient and sustained reductions in weight to reduce an individual’s risk of obesity-related morbidity and mortality [1, 2]. However, a large body of evidence suggests that bariatric surgery can result in sustained weight loss, reduce an individual’s risk of type 2 diabetes mellitus (T2DM) recurrence, and decrease levels of circulating inflammatory markers associated with obesity, the metabolic syndrome, and T2DM [3–5]. Therefore, bariatric surgery is adopted to an increasing extent globally as a treatment for morbid obesity and its secondary complications [6–8].

In recent years, a growing number of studies have begun to realise the potential of microvesicles (MVs) as biomarkers for a number of diseases, including the metabolic syndrome (MetSy) [9], T2DM [10], and atherosclerosis [11]. MVs are a subset of extracellular vesicles (EVs) that can be distinguished by their biogenesis, namely outwards budding of the plasma membrane and subsequent occlusion and shedding of the particle [12]. Like all other extracellular vesicles, MVs are a heterogeneous group of plasma membrane-enclosed particles containing components from their cellular origin, which are shed by most cell types in latent, activated and apoptotic states [13]. A soluble form of CD36 (sCD36) has previously been demonstrated to be present in circulation, and increased levels have been associated with abdominal fat distribution [14], insulin resistance [15], and non-alcoholic fatty liver disease (NAFLD) [16]. Although the exact nature of sCD36 is unknown, studies have previously revealed that, at least in part, sCD36 is associated with circulating MVs [11, 17].

CD36 is a scavenger receptor that has been associated with cellular uptake of lipids in a whole range of cells, and its expression is increased in obesity, MetSy, and diabetes [18–23]. In addition, a large body of evidence suggests that CD36 is involved in pro-inflammatory polarisation of macrophages upon exposure to oxidised LDL cholesterol and therefore contributes to the low-grade inflammatory state observed in diet-induced obesity and MetSy [22, 24]. Thus, measuring CD36 on the surface of MVs might not only yield important information about ectopic fat deposition but also the level of low-grade inflammation.

Therefore, it was hypothesised that significant weight loss and improvement in cardio-metabolic risk factors resulting from bariatric surgery would result in decreased concentrations of circulating MVs, MVs positive for the expression of CD36 on their surface, MVs of monocyte (MMVs) or endothelial (EMV) origin, and MMVs and EMVs positive for CD36. It was further hypothesised that subjects suffering from MetSy at inclusion had increased baseline concentrations of all of the abovementioned MV phenotypes.

## Methods

### Study design

The study population and design have been described elsewhere [14]. In brief, twenty individuals undergoing bariatric surgery were recruited for the present study. All participants met the Danish requirements for referral to bariatric surgery: ≥ 20 years of age and a body mass index (BMI) ≥ 40 kg m-2 or BMI ≥ 35 with associated co-morbidities. Participants were additionally required to lose approximately 8% of their body weight prior to surgery and inclusion in this study. Bariatric surgery was performed by either one of two surgeons with expertise in *Roux-en-Y* gastric bypass using a standard laparoscopic *Roux-en-Y* technique at the Aleris-Hamlet Hospital, Copenhagen, Denmark [14]. All participants were dismissed within 24 h after surgery and no subjects suffered from post-surgical complications.

All twenty subjects were evaluated prior to (baseline) and three months after bariatric surgery. At each visit, following an overnight fast, height and weight were recorded, fat mass and distribution was measured by full body dual-energy x-ray absorptiometry, and venous blood samples were collected for biochemical analyses and flow cytometric measurement of MVs. Haemoglobin, leukocytes, aspartate aminotransferase (AST), alanine aminotransferase (ALT), glycosylated haemoglobin, plasma glucose, C-peptide, serum insulin, total cholesterol, high-density lipoprotein, low-density lipoprotein, and triglycerides were determined with standardised methods in a routine biochemical laboratory. Plasma soluble CD36 (sCD36) was measured with an in-house ELISA as previously described [15]. Serum YKL40 was determined with a commercial ELISA (Quidel, San Diego, CA, USA). High sensitivity C-reactive protein (hsCRP) was measured with a commercial highly sensitive, latex particle-enhanced immunoturbidimetric assay (DAKO, Glostrup, Denmark). Furthermore, insulin sensitivity was determined with the homeostasis model assessment (HOMA-%S, http://www.dtu.ox.ac.uk/homacalculator/index.php), and liver fat percentage (LF%) was predicted using an algorithm based on the presence of MetSy and T2DM as well as fasting insulin and ALT and AST [25].

### Flow cytometric analysis of MVs

**Fig. 1:**
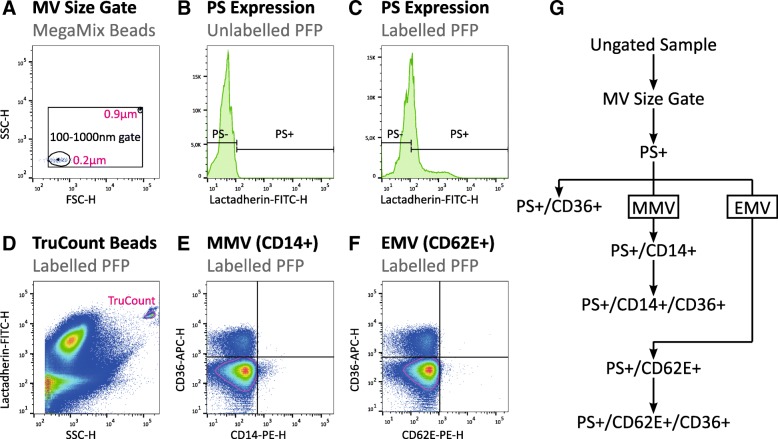
Gating strategy for flow cytometric characterisation of MVs. **a**) A 100-1000 nm MV size gate was established based on 200 nm and 900 nm polystyrene beads (MegaMix) and transferred to all samples. **b**) Next, a gate was set on FITC-H at the 99th percentile of unlabelled samples. **c**) MVs were defined as PS+ events based on binding of lactadherin-FITC. **e** & **f**) The double negative population was defined based on density (magenta gate), and bi-variate gates were placed at the 99th percentile of the double negative population. Finally, the gates were applied to all PS+ events in the corresponding sample to define MMVs (**e**), EMVs (**f**), and the expression of CD36 on these phenotypes (**e** & **f**). **d**) TruCount® beads (magenta gate) were quantified and used to calculate absolute concentrations of MVs. **g**) Gating hierarchy utilised for defining the MV phenotypes in the current study. PS: Phosphatidylserine; PFP: Platelet-free plasma; MMV: Monocyte microvesicles; EMV: Endothelial microvesicles

Blood samples for flow cytometric analysis of MVs were collected into EDTA tubes and the first centrifugation cycle was initiated within two hours after collection. Samples were subjected to centrifugation at 2000 g for 10 min to yield blood plasma. Plasma was stored at − 80 °C until analysis. Flow cytometric analysis of plasma MV content was performed as described previously [26] and in supplementary materials on a BD FACSAria™ III High Speed Cell Sorter (BD Biosciences, San Jose, CA, USA) and data was analysed in FlowJo® version 10.4 (FlowJo LLC, Oregon, USA) as depicted in Fig. 1.

### Statistical data analysis

All statistical data analyses and plotting was performed in and R 3.2.5 (R Core Team, Vienna, Austria) with *xlsx* [27], *ggplot2* [28], and *reshape2* [29] packages installed. The assumption of normality was tested using Shapiro-Wilk’s W-test and confirmed visually for each parameter using QQ-plots and histograms. Paired Student’s t-test or Wilcoxon signed rank test were used to compare pre and post-surgical values for all parameters where appropriate, whereas unpaired Student’s t-test or Mann-Whitney U test were utilized to compare baseline parameters with regards to the presence of MetSy on baseline data. All *P*-values are two-sided, and statistical significance was defined as *p* < 0.05.

## Results

### Anthropometric and biochemical characteristics

This study population has previously been described by Knøsgaard et al. [14] and data are presented in Table 1. In summary, 18 female and 2 male subjects were included into the current study, and subjects were 46.5 (range: 26; 63) years of age at inclusion. Subjects had a median weight of 118 kg (IQR: 108.75; 127) and BMI of 42.1 kg m^− 2^ (IQR: 40.4; 44.1). At the three-month post-surgical follow-up visit, subjects had incurred significant decreases in body weight (follow-up: 94 kg (IQR: 84.7; 100); *n* = 20; *p* < 0.0001) and BMI (follow-up: 33.9 kg m^− 2^ (IQR: 32.0; 35.0); *n* = 20; *p* < 0.0001).

**Table 1:** Characteristics of the study population

	Baseline	3 monthFollow-up	Δ	*P*-value
	(*n* = 20)	(*n* = 20)		
Age [Years]	46.5 ± 11.2			
Sex [M/F]	2/18			
Metabolic Syndrome [*n* (%)]	13 (65%)	2 (10%)		
NASH [*n* (%)]	13 (65%)	11 (55%)		
Haemoglobin [mmol l^−1^]	8.5 ± 0.5	8.4 ± 0.6	−0.1 ± 0.4	0.1405
Weight [kg]	118 (108.75; 127)	94 (84.7; 100)	−23.33 ± 4.21	**0.0001**
BMI [kg m^− 2^]	42.1 (40.4; 44.1)	33.9 (32.0; 35.0)	−8.4 ± 1.4	**< 0.0001**
Total Fat Mass [kg]	54.8 ± 11.0	40.2 (32.9; 42.3)	−15.6 ± 35. 6	**< 0.0001**
Body Fat Percentage [%]	45.3 ± 4.9	39.4 ± 6.5	−5.6 ± 2.4	**< 0.0001**
Fat Mass/Fat Free Mass [AU]	0.84 ± 0.16	0.67 ± 0.17	−0.16 ± 0.06	**< 0.0001**
Android Fat [kg]	48.2 ± 8.2	31.9 ± 9.6	−16.8 ± 4.7	**< 0.0001**
Android Fat Percentage [%]	46.8 ± 4.0	39.5 ± 6.0	−7.3 ± 3.8	**< 0.0001**
Truncal Fat [kg]	26.6 ± 40.3	17.9 ± 49.2	−8.4 ± 2.9	**< 0.0001**
Truncal Fat Percentage [%]	44.5 ± 3.7	37.5 ± 6.4	−6.6 ± 3.9	**< 0.0001**
Total Cholesterol [mmol l^−1^]	4.92 ± 1.00	4.49 ± 0.88	−0.44 ± 0.82	**0.0275**
HDL Cholesterol [mmol l^−1^]	1.14 ± 0.27	1.12 ± 0.28	−0.02 ± 0.15	0.659
LDL Cholesterol [mmol l^−1^]	2.99 ± 0.87	2.82 ± 0.69	−0.18 ± 0.73	0.2992
Oxidized LDL Cholesterol	4.53 ± 1.13	4.34 ± 0.93	−0.19 ± 0.84	0.3203
Triglycerides [mmol l^−1^]	1.72 ± 0.62	1.15 (1.05; 1.45)	−0.46 ± 0.52	**0.001**
Triglyceride/HDL [AU]	1.38 (1.09; 1.89)	1.03 (0.86; 1.41)	−0.41 ± 0.54	**0.0023**
Glycated Haemoglobin [%]	5.9 ± 0.43	5.6 (5.5; 5.8)	−0.14 ± 0.40	0.0792
Fasting Glucose [mmol l^−1^]	5.45 (5.18; 6.03)	5.1 (4.78; 5.33)	−0.45 (− 0.8; − 0.15)	**0.0157**
Fasting C-Peptide [pmol l^− 1^]	1061 (921; 1422)	745 (566; 935)	− 360 (− 542; −36.3)	**0.0094**
Fasting Insulin [pmol l^− 1^]	131.7 (97.5; 201.5)	63.7 (53.7; 77.8)	−69.7 (− 105.7; −40.8)	**0.0014**
HOMAIR	2.55 (2.05; 3.2)	1.6 (1.3; 2.05)	−0.85 (− 1.43; − 0.075)	**0.0149**
HOMASE	42.7 ± 16.4	60.4 ± 20.7	17.7 ± 26.3	**0.0072**
YKL40 [ng ml^−1^]	57.5 (47; 68.3)	58 (45; 72.8)	1.1 ± 14.8	1
Liver Fat Percentage [%]	6.7 (4.54; 9.99)	2.82 (2.23; 3.79)	−2.78 (−6.23; −1.84)	**0.0002**
ALT [U I^−1^]	30.9 ± 13.6	29.4 ± 15.8	−1.45 ± 20.4	0.7537
AST [U I^−1^]	28 (25; 32.3)	26 (22; 37)	0.65 ± 12.1	0.9826
AST/ALT [AU]	0.97 (0.88; 1.36)	0.97 (0.92; 1.24)	0.06 (−0.11; 0.30)	0.33
Leukocytes [mia l^−1^]	8.37 ± 2.24	6.65 (5.73; 8.08)	−1.34 ± 1.15	**0.0003**
High-sensitivity CRP [mg l^−1^]	4.67 (2.78; 9.99)	2.16 (1.28; 5.16)	−3.91 ± 6.15	**0.0073**
Soluble CD36 [AU]	0.48 ± 0.20	0.37 (0.24; 0.40)	−0.13 (−0.19; − 0.06)	**0.0008**

Data are depicted as mean ± SD or median (Q_25%_; Q_75%_)

*p*-values < 0.05 in bold text

### Effect of bariatric surgery on microvesicle phenotypes

**Fig. 2:**
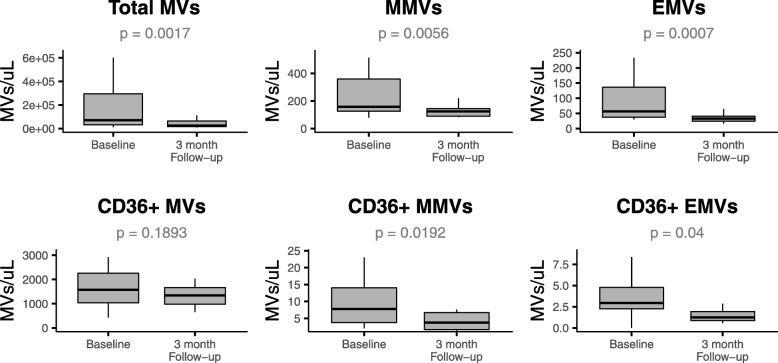
Baseline (*n* = 20) and three-month follow-up (*n* = 20) concentrations of MV phenotypes in 20 individuals. Significant decreases from baseline to follow-up were observed for PS+ MVs, MMVs, EMVs, and CD36+ MMVs and CD36+ EMVs, however no differences were observed in CD36+ MV concentration. Outliers are not depicted due to their extreme nature

In the present study, plasma MV content and phenotypic origin of EVs were determined by flow cytometry. At the three-month post-surgical follow-up visit, significant decreases were observed in all of the investigated MV phenotypes with the exception of CD36+ MVs, which remained unaltered (Fig. 2 and Additional file 1: Table S2).

Specifically, concentrations of phosphatidylserine positive (PS+) MVs decreased by a median of 66.55% from baseline to follow-up (*p* = 0.0017). This was equally accompanied by altered concentrations of MMV sub-phenotypes, where MMVs decreased by 36.11% (*p* = 0.0056) and CD36+ MMVs by 60.04% (*p* = 0.0192). Similar results were observed for EMV sub-phenotypes, where EMV concentrations decreased by 40.10% (*p* = 0.0007) and CD36+ EMVs by 54.93% (*p* = 0.04).

### Microvesicles and metabolic syndrome

At baseline, thirteen subjects were defined as having MetSy [30], while only two subjects were defined as having MetSy at the three-month post-surgical follow-up visit. In order to investigate the impact of MetSy on MV phenotypes, subjects were initially stratified into metabolically healthy (MH; *n* = 7) and metabolically unhealthy (MuH; *n* = 13) groups based on the presence of MetSy prior to surgery (characteristics presented in Additional file 1: Table S3), and baseline concentrations of MV phenotypes compared between the two groups. However, no significant differences could be observed in baseline concentrations of any of the investigated MV phenotypes between MH and MuH groups (Additional file 1: Table S3).

## Discussion

Previously, in the cohort of this study, where severely obese subjects underwent bariatric surgery, we investigated how weight loss secondary to bariatric surgery affects levels of circulating sCD36 [14]. In the present study on the other hand, we investigated how different MV phenotypes specifically focussing on MMVs, EMVs and their respective phenotypes positive for the expression of CD36 are affected by weight loss following bariatric surgery. In this study,, the results were two-fold. First, concentrations of total MVs, MMVs, EMVs, CD36+ MMVs and CD36+ EMVs decreased significantly following bariatric surgery. Second, and somewhat conversely, there were no differences in baseline concentrations of any of the investigated MV phenotypes between MH and MuH subjects.

In the present study, the concentration of PS+ MVs was found to decrease significantly following bariatric surgery. In contrast to the present study, Witczak et al. described that the total number of extracellular vesicles were unaltered following bariatric surgery [31]. However, this discrepancy could be due to different pre-analytical methodology and different methods for characterising EVs, which might result in a different subset of EVs being analysed. It has previously been established that platelet MVs are the most abundant phenotype of MVs present in plasma [32], and that platelet MVs are up-regulated in obese subjects [33, 34]. Levels of platelet MVs seem to correlate with body composition [33], plasma lipids [35], and hyperglycaemia [36], all attributing to oxidative stress [37]. These parameters also seem to affect several other MV phenotypes including leukocyte MVs [38, 39], EMVs [33], and MMVs [35]. Thus, it can be inferred that the improvements observed in body composition, plasma lipid profile, and insulin sensitivity result in decreased activation of platelets, leukocytes, vascular endothelial cells, and monocytes/macrophages, which in turn could explain the significant reduction in PS+ MVs.

An interesting observation in the present study was that CD36+ MVs did not differ significantly from baseline to the three-month follow-up. This result is in contrast to a study conducted by Campello et al., in which CD36+ MVs decreased significantly from baseline to three and twelve-months follow-up visits [39]. Interestingly, although CD36+ MVs were unaltered, a significant decrease was seen in sCD36 in the present cohort. Levels of sCD36 have previously been suggested to reflect those of tissue expression [15] and further associated with unhealthy fat distribution [14], insulin resistance [15], and hepatic fat accumulation [16], all complications of obesity. It can thus be argued that sCD36 might be a more sensitive marker for cardio-metabolic complications than MV-associated CD36.

Bariatric surgery led to reductions of MMVs and CD36+ MMVs in the present study. In addition to demonstrating that bariatric surgery reduces MMVs, Cheng et al. also demonstrated that MMVs were associated with BMI and HbA1c [40]. CD36+ MMVs were further demonstrated to correlate with BMI, waist circumference, total fat mass, triglycerides, and fasting levels of C-peptide and insulin in another study [35]. A growing body of evidence is starting to recognise that macrophages play a central role in the development of insulin resistance and T2DM (reviewed in [41]). In obese individuals, steatotic cells secrete chemokines that promote migration of monocytes into tissues and their subsequent polarisation into a more pro-inflammatory phenotype [42, 43]. Apart from this, a growing body of evidence has implicated CD36 on the surface of macrophages and its ability to interact with oxidised LDL cholesterol in the pro-inflammatory polarisation of macrophages [22]. Moreover, the expression of CD36 on the surface of macrophages is increased in obese individuals [44]. Thus, it is possible that the significant weight loss observed following bariatric surgery and the concurrent improvements in cardio-metabolic health leads to decreased recruitment of monocytes to steatotic tissues, increased proportion of anti-inflammatory macrophages, and overall decreased activity in macrophages. This could in turn lead to a reduction in the release of MMVs and CD36+ MMVs.

EMVs and CD36+ EMVs were found to be significantly reduced following bariatric surgery in the current study. Studies that have previously examined the impact of bariatric surgery on EMV concentrations have yielded conflicting results. In line with the present study, both Campello et al. [39] and Cheng et al. [40] reported that levels of various phenotypes of MVs including platelet MVs, EMVs and MMVs decreased significantly in obese subjects after bariatric surgery. Conversely, Stepanian et al. [34] reported no differences in platelet MVs and EMVs one year after subjects had undergone bariatric surgery, which was later supported by a study conducted by Witczak et al. [31]. However, a growing body of evidence is in support of there being a relationship between EMVs and obesity, MetSy, and T2DM [33, 45–48]. Ectopic fat deposition and hyperglycaemia have long been known to contribute to endothelial dysfunction and subsequent atherosclerosis by means of dysregulation of circulating lipids [49]. A common feature of endothelial dysfunction is the production of ROS resulting from altered intracellular metabolism [50], which in turn leads to mitochondrial fragmentation, increased endothelial permeability, and up-regulation of adhesion molecules and pro-inflammatory cytokines [51]. In turn, this promote the attachment, migration and polarisation of monocytes into underlying tissues and a subsequent inflammatory response. Some doubt has to be cast on the concentrations of EMV sub-phenotypes due to similar binding patterns of the EMV antibody and its isotype control utilised in the current assay (Additional file 2: Figure S1). It can therefore not be ruled out that the current results could have arisen from non-specific binding of the antibody and subsequent inclusion of false positive events. However, the stark differences between baseline and the three-month follow-up visit are striking nonetheless.

Somewhat controversially, baseline concentrations of MV phenotypes did not differ between subjects with and without MetSy. This result is in support of observations by Stepanian et al. [34], who also observed no differences in levels of platelet MVs and EMVs between metabolically healthy subjects and subjects with MetSy. On the other hand, Chironi et al. [38] reported that leukocyte MVs were higher in individuals with MetSy in a non-obese cohort, however EMVs did not differ between groups in their study. Conversely, Amabile et al. [47] and Arteaga et al. [52] demonstrated that several phenotypes of EMVs were associated with the MetSy and correlated with number of components of MetSy. Although it was hypothesised that MuH subjects had increased levels of the investigated MV phenotypes, the authors recognise that several confounding factors might have influenced the results of the present study including BMI and body composition. To a certain extent explain, this might also differences observed between studies, as mean BMI differs greatly between studies.

A significant strength of the current study is that it employs flow cytometry to quantify and characterise MVs from subjects undergoing bariatric surgery. Although conventional flow cytometry lacks the sensitivity to measure the smallest of extracellular vesicles [53], it remains a preferred method of characterising MVs and holds great advantages over a multitude of other methods for characterising and quantifying MVs including nano-particle tacking analysis, Western blot, and ELISA. This is due to its ability to simultaneously quantify MVs and characterise their expression of multiple surface markers in a high-throughput manner, thus allowing for discrimination between large numbers of MVs with different phenotypic origins [26, 54–56]. Several limitations should, however, also be addressed in the present study. First, the authors recognise that the sample size in the present study is a limitation, which could give the study insignificant power to discover significant differences in baseline concentrations of MVs between MH and MuH subjects. In addition, this could explain the lack of correlations between changes of MV phenotypes on the one hand and body composition, plasma lipids, insulin sensitivity, and inflammatory markers on the other (data not shown). Second, the follow-up in this study is relatively short, and it is therefore impossible to make inferences about the long-term effects of bariatric surgery on levels of circulating MVs. Third, due to constraints, isotype controls were prepared for baseline samples only, thus limiting the interpretability of flow cytometric characterisation of MVs in samples from follow-up visits, as the extent of non-specific binding of antibodies to MVs cannot be examined. Nonetheless, the magnitude of decrease in MV concentrations from baseline to follow-up would arguably imply that the importance of this is minimal if not insignificant.

## Conclusion

In conclusion, this study reports that concentrations of MVs and specifically those of monocyte and endothelial origin decrease significantly in the follow-up period following bariatric surgery. Moreover, the concentration of the bulk of CD36+ MVs remained unaltered, while significant decreases are seen in sCD36, CD36+ MMVs, and CD36+ EMVs. These changes likely reflect the significant weight loss incurred by the subjects following bariatric surgery, which in turn leads to reduction in ectopic deposition of fat, improved plasma lipid profile, decreased low-grade inflammation, and oxidative stress. At the cellular level, decreased amount of lipids in the extracellular space reduce CD36-specific uptake of lipids by endothelial cells and macrophages, which in turn reduces cell stress and CD36 expression on the cell membrane, thereby resulting in decreased shedding of MVs. However, baseline levels of MV phenotypes did not differ between subjects with MetSy and those without MetSy.

Finally, in order to further investigate the potential of MMVs, EMVs and their respective phenotypes positive for the expression of CD36, larger studies have to be conducted in which associations between their concentration and components of the metabolic syndrome and type 2 diabetes are thoroughly investigated.

## Additional files

Additional file 1: Supplementary methodological information on MV characterisation by flow cytometry and antibody panels used in this study. **Table S1.** Antibodies and concentrations used for flow cytometric characterisation of MVs. **Table S2.** Baseline and three-month follow-up concentrations of MV phenotypes in study participants. **Table S3.** Characteristics of the metabolically healthy and metabolically unhealthy participants. (DOCX 25 kb)

Additional file 2: **Figure S1.** Typical scatter plots for the two different antibody panels used to characterise MV phenotypes. (EPS 1189 kb)

## Abbreviations

ALT: alanine aminotransferase
AST: aspartate aminotransferase
BMI: Body mass index
CD: Cluster of differentiation
EMV: Endothelial-derived microvesicle
EV: Extracellular vesicle
HDL: High density lipoprotein
HOMA-%S: homeostasis model assessment of sensitivity
hsCRP: Highly sensitive C-reactive protein
IQR: Inter-quartile range
LDL: Low density lipoprotein
LF%: liver fat percentage
MetSy: Metabolic syndrome
MH: Metabolically healthy
MMV: Monocyte-derived microvesicle
MuH: Metabolically unhealthy
MV: Microvesicle
NAFLD: Non-alcoholic fatty liver disease
PS: phosphatidylserine
sCD36: Soluble CD36
T2DM: Type 2 diabetes mellitus
