# Supplementary material for: Bariatric surgery reduces CD36-bearing microvesicles of endothelial and monocyte origin
Source: Nutr Metab (Lond). 2018 Oct 23;15:76. doi: 10.1186/s12986-018-0309-4 (PMC6199798; doi:10.1186/s12986-018-0309-4)
Supplement: Supplementary file 1 — Supplementary methodological information on MV characterisation by flow cytometry and antibody panels used in this study. Table S1. Antibodies and concentrations used for flow cytometric characterisation of MVs. Table S2. Baseline and three-month follow-up concentrations of MV phenotypes in study participants. Table S3. Characteristics of the metabolically healthy and metabolically unhealthy participants. (DOCX 25 kb) [file 12986_2018_309_MOESM1_ESM.docx]

# Supplementary Methods

## Flow cytometric analysis of MVs

50µL of freshly thawed plasma was transferred to TruCount® tubes (BD Biosciences, San Jose, CA, USA) in order to allow quantitation of measured events. Samples were initially incubated with 10µL Fc receptor blocking reagent (BD Biosciences) for 10 minutes at 4°C. This was followed by incubation for 30 minutes at 4 °C in the dark with either of two separate antibody panels or matching isotype controls as described in supplementary table S1. After incubation, samples were diluted with 200µL Dulbecco’s Phosphate Buffered Saline 0.0095 M PO_4_ (PBS) buffer (Lonza, Basel Switzerland) that had been filtered through a sterile 0.2 µm Q-Max syringe filter (Frisenette, Knebel, Denmark). Flow cytometric analysis of plasma MV content was performed as described previously[26] on a BD FACSAria™ III High Speed Cell Sorter (BD Biosciences) with a triggering threshold set at 200 on side scatter peak intensity (SSC-H) to avoid exclusion of small particles. Samples were analysed at a maximal rate of 2 x 10^4^ events per second until 10^6^ events were collected in total.

## Analysis of flow cytometry data

Gating and extraction of statistics from flow cytometry data was conducted as depicted in figure 1 in FlowJo® version 10.4 (FlowJo LLC, Oregon, USA). First, a size gate spanning from 100nm to 1000nm was created on a bi-variate scatter plot of log-scaled forward scatter peak intensity (FSC-H) versus log-scaled SSC-H using a percentile gate (0.01^th^ and 99.99^th^ percentiles on both axes) around 200nm and 900nm fluorescent, size calibrated beads (Megamix, Biocytex, Marseille, France) and applied to all samples. Next, a gate was set on log-scaled FITC fluorescence peak intensity (FITC-H) at the 99^th^ percentile of unlabelled samples, and MVs were defined as phosphatidylserine positive (PS+) events based on binding of lactadherin-FITC in corresponding samples. Finally, MV phenotype gates were defined in bi-variate scatter plots of log-scaled PE fluorescence peak intensity (PE-H) and log-scaled APC fluorescence peak intensity (APC-H) for each sample by: 1) defining the double negative population based on density; 2) creating bi-variate gates on the 99^th^ percentile of the double negative population; and 3) applying the gates to all PS+ events in the sample. MVs of monocyte origin (MMVs) were defined as CD14 positive events, endothelial MVs (EMVs) were defined as CD62E positive events, and the expression of CD36 was investigated on all PS+ MVs, MMVs and EMVs. Total MV concentration and CD36+ MV concentration was calculated as the average for each sample across the two antibody panels. MV concentration was calculated based on the quantity of TruCount® beads collected in each sample by the following formula:

$$MV Concentration=\frac{MVs collected}{TruCount beads collected}\times\frac{TruCount beads in tube}{Sample volume}\times Dilution factor$$

**Table S1:** Antibodies and concentrations used for flow cytometric characterisation of MVs.

|  | **Antibody** | **Isotype control** |
| --- | --- | --- |
| **Panel 1** | **5µL Lactadherin-FITC** (83 μg/mL; Hematologic Technologies Inc., VT, USA) | **Blank** |
|  |  |  |
|  | **10µl CD14-PE** (60µg/mL; Clone TÜK-4 (IgG2a); DAKO, Denmark) | **3µL IgG2a-PE** (200µg/mL; DAKO, Denmark) |
|  |  |  |
|  | **15µl CD36-APC** (12.5µg/mL; Clone CB38 (IgM, κ); BD Pharmingen, New Jersey, USA) | **7.5µL IgM-APC** (25µg/mL; BD Pharmingen, New Jersey, USA) |
|  |  |  |
|  |  |  |
| **Panel 2** | **5µL Lactadherin-FITC** (83 μg/mL; Hematologic Technologies Inc., VT, USA) | **Blank** |
|  |  |  |
|  | **10µl CD62E-PE** (100µg/mL; Clone 68-5H11 (IgG1, κ); BD Pharmingen, New Jersey, USA) | **20µL IgG1, κ-PE** (50µg/mL; BD Pharmingen, New Jersey, USA) |
|  |  |  |
|  | **15µl CD36-APC** (12.5µg/mL; Clone CB38 (IgM, κ); BD Pharmingen, New Jersey, USA) | **7.5µL IgM-APC** (25µg/mL; BD Pharmingen, New Jersey, USA) |
|  |  |  |

**Table S2:** Baseline and three-month follow-up concentrations of MV phenotypes in study participants.

|  | **Baseline** | **3 Month Follow-up** | ***P*-Value** |
| --- | --- | --- | --- |
|  | (*n* = 20) | (*n* = 20) |  |
| **Total MVs [µL^-1^]** | 71239 (31808; 295183) | 24193.25 (16647; 63922) | **0.0017** |
| **CD36^+^ MV [µL^-1^]** | 1577 (1040; 2258) | 1343 (982; 1664) | 0.1893 |
| **MMVs [µL^-1^]** | 157 (126; 359) | 124 (89.4; 144) | **0.0056** |
| **CD36^+^ MMVs [µL^-1^]** | 7.79 (3.75; 14.05) | 3.75 (1.70; 6.73) | **0.0192** |
| **EMVs [µL^-1^]** | 56.6 (37.2; 136.1) | 32.8 (24.5; 41.0) | **0.0007** |
| **CD36^+^ EMV [µL^-1^]** | 2.94 (2.25; 4.77) | 1.23 (0.87; 1.93) | **0.04** |

Data are depicted as median (Q_25%_; Q_75%_).

**Table S3:** Characteristics of the metabolically healthy and metabolically unhealthy participants.

|  | **Metabolically healthy** | **Metabolically unhealthy** | ***P*-Value** |
| --- | --- | --- | --- |
|  | (*n* = 7) | (*n* = 13) |  |
| **Sex [M/F]** | 0/7 | 2/11 |  |
| **Age [Years]** | 44.9±10.9 | 47.4±11.7 | 0.6368 |
| **Systolic Blood Pressure [mmHg]** | 120 (115; 120) | 121.9±16.8 | 0.7145 |
| **Diastolic Blood Pressure [mmHg]** | 75.7±6.7 | 80 (75; 80) | 0.4583 |
| **Pulse [minute^-1^]** | 80 (77; 86) | 88 (88; 88) | 0.5883 |
| **Haemoglobin [mmol l^-1^]** | 8.6±0.4 | 8.4±0.6 | 0.4455 |
| **Weight [kg]** | 113±12.6 | 120 (109; 127) | **0.0001** |
| **BMI [kg m^-2^]** | 41.7±3.5 | 43.6±6.1 | 0.3826 |
| **Total Fat Mass [kg]** | 54.6±9.0 | 54.9±12.4 | 0.9424 |
| **Body Fat Percentage [%]** | 47.5±4.1 | 44.2±5.0 | 0.1332 |
| **Fat Mass/Fat Free Mass [AU]** | 0.91±0.14 | 0.80±0.15 | 0.1321 |
| **Android Fat [kg]** | 4.82±0.78 | 4.82±0.89 | 0.9957 |
| **Android Fat Percentage [%]** | 48.7±3.7 | 45.6±3.9 | 0.1106 |
| **Truncal Fat [kg]** | 25.7±3.7 | 27.1±4.2 | 0.4677 |
| **Truncal Fat Percentage [%]** | 46.1±3.8 | 43.6±3.5 | 0.1783 |
| **Total Cholesterol [mmol l^-1^]** | 5.22±1.02 | 4.75±0.99 | 0.3352 |
| **HDL Cholesterol [mmol l^-1^]** | 1.30±0.26 | 1.05±0.23 | 0.0562 |
| **LDL Cholesterol [mmol l^-1^]** | 3.16±0.88 | 2.90±0.88 | 0.5447 |
| **Oxidized LDL Cholesterol** | 4.76±1.30 | 4.41±1.07 | 0.5516 |
| **Triglycerides [mmol l^-1^]** | 1.66±0.75 | 1.75±0.57 | 0.7711 |
| **Triglyceride/HDL [AU]** | 1.29±0.55 | 1.76±0.74 | 0.1274 |
| **Glycated Haemoglobin [%]** | 5.71±0.25 | 6.00±0.48 | 0.0792 |
| **Fasting Glucose [mmol l^-1^]** | 4.97±0.31 | 6.12±0.88 | **0.0157** |
| **Fasting C-Peptide [pmol l^-1^]** | 1110±275 | 1086 (937; 1420) | **0.0094** |
| **Fasting Insulin [pmol l^-1^]** | 136.6±61.4 | 139.7 (98.9; 200.1) | **0.0014** |
| **HOMAIR** | 2.44±0.59 | 2.7 (2.2; 3.2) | **0.0149** |
| **HOMASE** | 43.2±10.1 | 42.4±19.4 | 0.8989 |
| **YKL40 [ng ml^-1^]** | 45.1±16.3 | 66 (54; 69) | 1 |
| **Liver Fat Percentage [%]** | 4.43±0.82 | 9.81±4.83 | **0.0017** |
| **ALT [U I^-1^]** | 34.6±14.0 | 28.8±13.5 | 0.3946 |
| **AST [U I^-1^]** | 31.9±9.4 | 28.9±7.8 | 0.4954 |
| **AST/ALT [AU]** | 0.99±0.28 | 1.15±0.42 | 0.33 |
| **Leukocytes [mia l^-1^]** | 8.03±2.31 | 8.55±2.28 | 0.6351 |
| **High-sensitivity CRP [mg l^-1^]** | 7.72±6.91 | 4.12 (2.82; 9.823) | 1 |
| **Soluble CD36 [AU]** | 0.37±0.14 | 0.53±0.21 | 0.0624 |
| **Total MVs [µL^-1^]** | 60593 (31734; 154426) | 127850 (47838; 440341) | 0.3507 |
| **CD36^+^ MV [µL^-1^]** | 1872±952 | 1265 (1124; 1917) | 0.4378 |
| **MMVs [µL^-1^]** | 138 (132; 188) | 242 (120; 376) | 0.5356 |
| **CD36^+^ MMVs [µL^-1^]** | 8.38 (5.07; 16.02) | 7.19 (3.72; 13.3) | 0.7573 |
| **EMVs [µL^-1^]** | 44.0 (37.9; 85.5) | 66.2 (37.1; 143.1) | 0.5356 |
| **CD36^+^ EMV [µL^-1^]** | 2.96±1.68 | 4.68±3.65 | 0.1682 |

Data are depicted as mean±SD or median (Q_25%_; Q_75%_).

**Figure S1:** Typical scatter plots for the two different antibody panels used to detect MMVs (**A-C**) and EMVs (**D-F**).
